# Tuberculosis in elderly Australians: a 10-year retrospective review

**DOI:** 10.5365/wpsar.2024.15.1.1040

**Published:** 2024-01-05

**Authors:** Yasmin Lisson, Aparna Lal, Ben J Marais, Anna Glynn-Robinson

**Affiliations:** a National Centre for Epidemiology and Population Health, College of Health and Medicine, Australian National University, Canberra, Australian Capital Territory, Australia.; b Office of Health Protection and Response Division, Australian Government Department of Health and Aged Care, Canberra, Australian Capital Territory, Australia.; c Centre for Research Excellence in Tuberculosis, University of Sydney, Sydney, New South Wales, Australia.; d Marie Bashir Institute for Infectious Diseases and Biosecurity, University of Sydney, Sydney, New South Wales, Australia.

## Abstract

**Objective:**

This report describes the epidemiology of active tuberculosis (TB) in elderly Australians (≥ 65 years) with analysis of the factors associated with TB disease and successful treatment outcomes.

**Methods:**

A retrospective study of TB cases reported to the National Notifiable Diseases Surveillance System over a 10-year period from 2011 to 2020 was conducted. Cases were stratified by sex, age, risk factors, drug resistance, treatment type and outcome. Notification rates and incidence rate ratios with 95% confidence intervals were calculated and factors associated with treatment success analysed using multivariable logistic regression.

**Results:**

A total of 2231 TB cases among elderly people were reported over the study period, with a 10-year mean incidence rate of 6.2 per 100 000 population. The median age of cases was 75 years (range 65–100 years); most were male (65%) and born overseas (85%). Multivariable analysis found that successful treatment outcome was strongly associated with younger age, while unsuccessful treatment outcome was associated with being diagnosed within the first 2 years of arrival in Australia, ever having resided in an aged-care facility and resistance to fluoroquinolones.

**Discussion:**

Compared to other low-incidence settings in the Western Pacific Region, TB incidence in elderly people is low and stable in Australia, with most cases occurring among recent migrants from TB-endemic settings. Continued efforts to reduce TB importation and address migrant health, especially among elderly people, are important.

The Western Pacific Region, including Australia, is home to over 1.9 billion people across 27 countries and 10 areas. (*1*, *2*) The Region has undergone rapid demographic change in recent years, with declining fertility and increasing life expectancy, (*1*) and is now home to the largest and fastest-growing ageing population in the world. There are currently more than 240 million elderly people (aged ≥ 65 years) living within the Region, with population numbers expected to increase twofold by 2050. (*2*, *3*) The World Health Organization (WHO) Regional Office for the Western Pacific is prioritizing a large Healthy Ageing initiative through multisectoral action, which includes reducing tuberculosis (TB) among elderly people. (*2*)

TB is caused by the bacterium *Mycobacterium tuberculosis* complex and is a leading cause of morbidity and mortality worldwide. (*4*, *5*) It is estimated that a quarter of the world’s population is infected with TB bacteria, (*5*) also referred to as latent TB infection (LTBI), (*6*) but disease occurs when a person develops clinical manifestations such as coughing, fever, weight loss and night sweats. (*6*, *7*) LTBI is an asymptomatic condition that cannot be transmitted to others, (*8*) although 5–15% of people living with LTBI are at risk of future progression to TB disease due to host, environmental and social risk factors. (*8*) Recognized TB risk factors include age, immunosuppression, alcohol and illicit drug use, smoking tobacco, malnutrition, diabetes mellitus, health-care work and incarceration. (*9*)

The Western Pacific Region has one of the highest TB disease burdens globally. (*5*) In TB-endemic settings with high rates of ongoing transmission, disease incidence rates are highest in younger adults (< 50 years). (*5*) However, in lower-incidence settings with declining rates of TB transmission, disease incidence rates among elderly people (aged ≥ 65 years) are often highest. (*5*) This is mainly due to increased comorbidity and immune dysfunction associated with increasing life expectancy and high rates of LTBI in older people who are at risk of reactivation of disease, together with reduced transmission and disease rates in the general population. (*10*)

Australia has sustained low incidence rates of TB over the last four decades. (*11*) However, due to most of cases occurring among recent migrants from high-incidence countries, elimination of TB continues to pose clinical and epidemiological challenges. Between 2011 and 2020, the absolute number of TB cases in Australia increased by 16%, in line with population growth (*11*) that includes a high proportion of people who were born in countries with high TB incidence, including China, India, Indonesia, Nepal and the Philippines. (*12*) Although Australia employs stringent pre-migration screening measures, this only includes screening for active pulmonary TB disease. (*13*) Most cases are identified in the first 5 years after arrival in Australia, representing likely disease reactivation. (*11*, *14*)

To date, there are few published reports outlining the epidemiology, risk factors and outcomes associated with TB in elderly people, despite accounting for close to 20% of all cases in Australia. (*11*) This study will address the current knowledge gap by describing the epidemiology of TB in elderly Australians and examining factors associated with it and successful treatment outcomes.

## Methods

Confirmed TB notifications (*15*) received between 1 January 2011 and 31 December 2020 were extracted in July 2022 from the National Notifiable Diseases Surveillance System (NNDSS). Data were stratified into two age group categories, elderly (≥ 65 years) and non-elderly (≤ 64 years), and were analysed using Stata/SE 17.0 (StataCorp, College Station, United States of America) and Excel (Microsoft Corporation, Redmond, United States of America). Australian Bureau of Statistics (ABS) mid-year resident population estimates were used to calculate annual notification rates per 100 000 population by age group, sex and country of birth. Incidence rate ratios (IRRs) were calculated with associated 95% confidence intervals (CIs) and *P*-values for 5-year age groups and sex. The methodology outlined in a previous national TB analysis (*16*) was followed, in which categorical data on “TB treatment outcome” were aggregated into four outcomes for descriptive analysis and binary outcomes for inferential analysis (**Box 1**). Crude odds ratios (ORs) and *P*-values were calculated to determine if demographic details, clinical symptoms, treatment regimens and drug resistance profiles were associated with treatment success using univariate logistic regression. Variables that were statistically significant (*P* < 0.05) in the univariate analysis were included in multivariable logistic regression. This model controlled for the effects of age, resistance to fluoroquinolones, ever having resided in an aged-care facility, and time from arrival to TB diagnosis (0–2 years), using backward stepwise elimination at a 0.05 significance level to create a final reduced model. Migrants were defined as those born overseas.

**Box 1:**
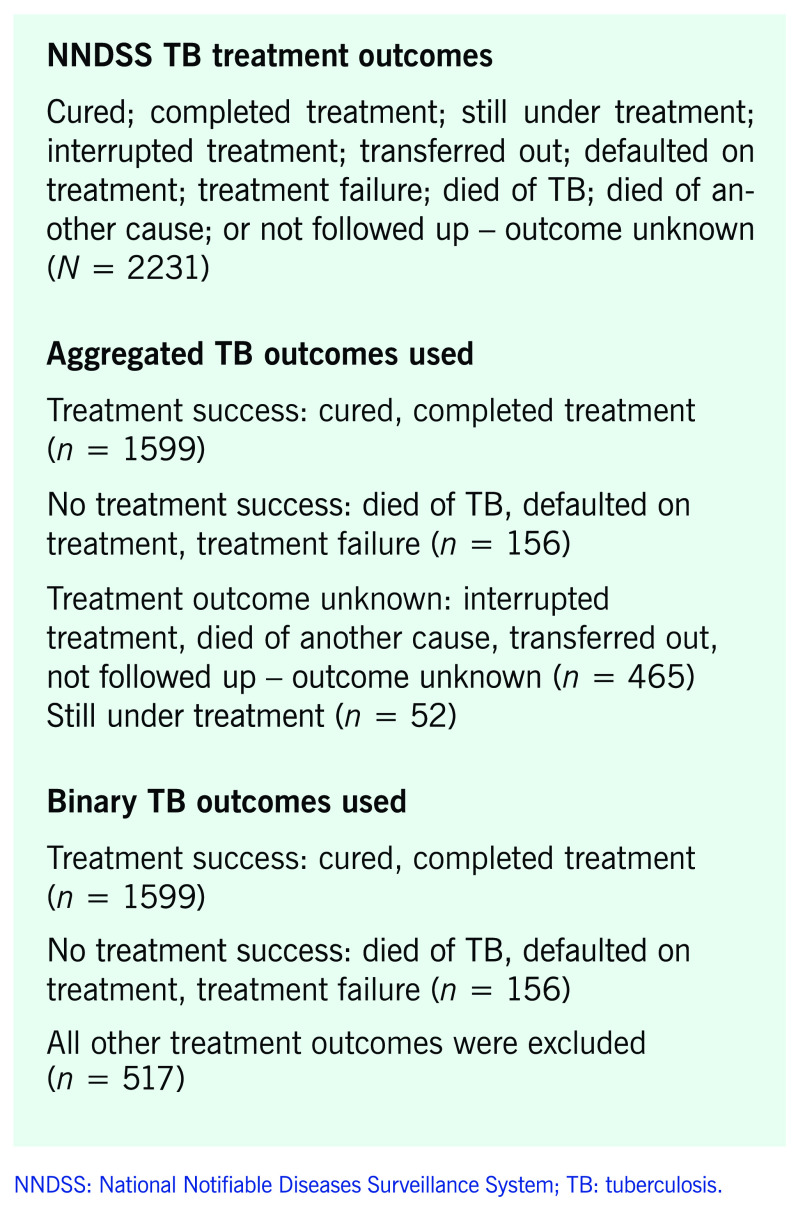
TB treatment outcomes extracted from the Australian NNDSS, 1 January 2011 to 31 December 2020

[**insert** Box 1]

## Results

During the study period, 13 917 cases were notified, of which 11 686 (83.9%) were aged ≤ 64 years and 2231 (16.1%) were aged ≥ 65 years, with average notification rates of 5.6 per 100 000 population and 6.2 per 100 000 population, respectively. The mean number of notifications in elderly people remained low, with a non-significant increase when comparing the reporting periods 2011–2015 (*n* = 197) and 2016–2020 (*n* = 249).

Australian-born TB cases represented 10.5% of non-elderly and 15.1% of elderly cases. Elderly cases reported their country of birth in the following order of frequency: Australia, China, Viet Nam, India and the Philippines (**Table 1**). Compared with non-elderly, elderly cases experienced more treatment failure (2.2% vs 6.9%, respectively) and unknown treatment outcome (8.1% vs 19.0%, respectively).

**Table 1:** Notifications of TB in Australians aged ≥ 65 years by selected demographic characteristics and treatment outcomes, Australia, 2011–2020

Characteristics	*n*(%) *n* = 2 231
**Age group**	
65–69 years	556 (24.9)
70–74 years	481 (21.6)
75–79 years	433 (19.4)
80–84 years	350 (15.7)
≥ 85 years	411 (18.4)
**Country of birth**	
Australia	336 (15.1)
China (excludes Hong Kong Special Administrative Region SAR, Macao SAR and China, Taiwan)	312 (14.0)
Viet Nam	275 (12.3)
India	175 (7.9)
Philippines	148 (6.6)
Cambodia	72 (3.2)
Other	911 (40.9)
Unknown	2 (0.09)
**On a TB health undertaking^a^ at time of diagnosis (yes)**	78 (3.5)
**Treatment outcomes**	
Treatment success^b^	1599 (71.7)
Treatment outcome unknown^c^	424 (19.0)
No treatment success^d^	156 (6.9)
Still under treatment	52 (2.3)
Died of TB^e^	109 (4.9)

SAR: Special Administrative Region; TB: tuberculosis.

^a^ A TB health undertaking refers to an individual who completed an agreement with the Australian Government to meet the health requirement in relation to TB at the time of diagnosis. This applies to individuals who have a significant health condition and had their health examinations outside Australia or applied for a protection visa.

^b^ Treatment success refers to a case who is cured of TB and completed treatment for TB.

^c^ Treatment outcome unknown refers to interrupted treatment, cases who died of another cause, transferred out, or were not followed up – outcome unknown.

^d^ No treatment success refers to cases who died of TB, defaulted on treatment, or treatment failure.

**^e^ In Australia, the cause of death in relation to TB varies among experienced TB clinicians and public health officers, using representative TB death scenarios.** (*17*)

**Fig. 1:**
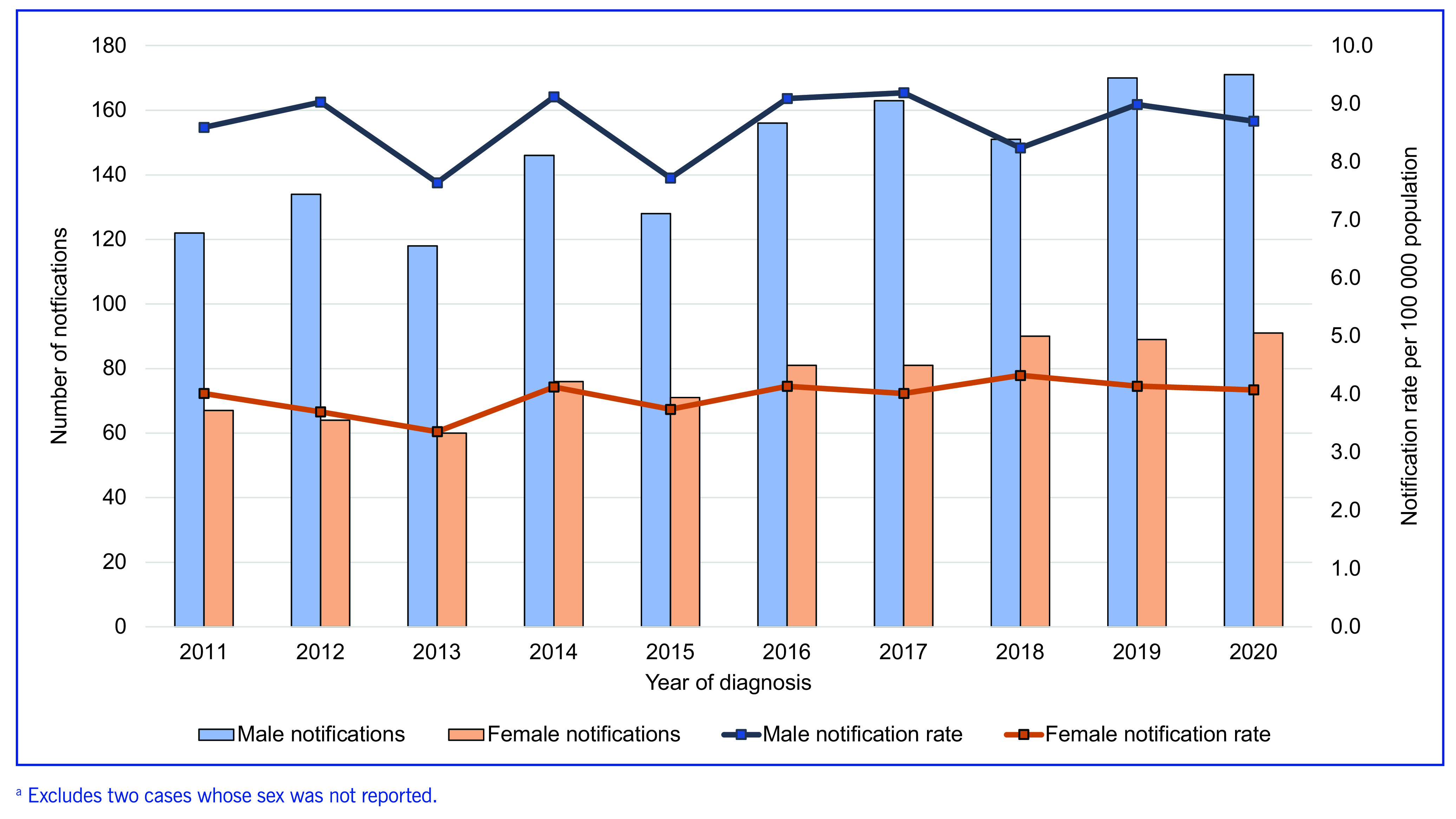
Sex-specific tuberculosis notification rates in elderly Australians, 2011–2020^a^

Over the 10-year period, there were consistent sex-specific differences in TB epidemiology, with significantly more men than women developing TB (IRR: 2.17 [95% CI: 1.64–2.90]; *P* < 0.05) (**Fig. 1**). Among elderly cases, the median age was 75 years (range: 65–100 years), with the highest notification rates observed in the 65–69-year age group. The majority (84.6%, *n* = 1887) of elderly cases were born overseas, and a small proportion (1.5%, *n* = 33) identified as Aboriginal and Torres Strait Islander people. Most elderly cases (91.7%) were classified as new, with 7.4% classified as relapse/recurrent cases (**Table 2**). Of those classified as relapse/recurrence, 65.2% had received full or partial treatment overseas, compared to 34.7% who had received full or partial treatment in Australia. Pulmonary TB was reported in 75.4% of cases, and only extra-pulmonary TB was reported in 24.4% of cases (**Table 2**). The most frequently reported risk factors documented among elderly TB cases included past travel to or residence (for at least 3 months cumulatively anytime in their life) in a country with high TB incidence (74.1%), having a household or other close contact with active TB (10.9%), and currently receiving immunosuppressive therapy (8.5%) (**Table 2**). Elderly overseas-born cases had a median time of 25 years (interquartile range [IQR]: 9–38 years) between arrival in Australia and their TB diagnosis. Among Australian-born cases, the median time from their initial health presentation to diagnosis was longer (44 days; IQR: 16–102 days), compared with overseas-born cases (31 days; IQR: 13–70 days).

[**insert** Figure 1]

**Table 2:** Characteristics of TB notifications in Australians aged ≥ 65 years, Australia, 2011–2020

Characteristics	*n*(%) *n* = 2 231
**Case classification**	
New^a^	2047 (91.7)
Relapse^b^	164 (7.4)
Unknown	20 (0.9)
**Case detection method**	
Clinical	1827 (81.9)
Screening	100 (4.5)
Contact tracing/epidemiological investigation	15 (0.7)
Unknown	289 (12.9)
**Diagnostic site (anatomical site)**	
Pulmonary TB^c^	1681 (75.4)
Extra-pulmonary TB only^d^	544 (24.4)
Unknown site of disease	6 (0.3)
**HIV testing**	
Positive	9 (0.4)
Negative	1398 (62.7)
Unknown	824 (36.9)
**Confirmed TB**	
Bacteriologically confirmed^e^	2021 (90.6)
Culture-confirmed	1789 (88.5)
Drug susceptibility testing^f^	1756 (98.2)
Clinically confirmed only	210 (9.4)
**Risk factors for TB^g^**	
Past travel to or residence in a country with high TB incidence^h^	1550 (74.1)
Household or other close contact with TB	228 (10.9)
Currently receiving immunosuppressive therapy	177 (8.5)
Chest X-ray suggestive of old untreated TB	125 (6.0)
Employed in the health industry in Australia or overseas, currently or in the past 5 years	73 (3.5)
Resided in an aged-care facility within the past 5 years	48 (2.3)
Resided in a correctional facility within the past 5 years	13 (0.6)
Homeless within the past 5 years	13 (0.6)
Employed at an aged-care facility, correctional facility or homeless shelter within the last 5 years	7 (0.3)
**Total cases assessed for risk factors**	**2092 (93.8)**
**Drug susceptibility profile^i^**	
Fully susceptible	1553 (85.6)
Resistance to at least one first-line anti-TB agent^j^	168 (9.3)
Mono-resistance to isoniazid	77 (4.2)
Mono-resistance to rifampicin	6 (0.3)
Resistance to at least one second-line injectable anti-TB agent^k^	3 (0.2)
Resistance to fluoroquinolones^l^	4 (0.2)
MDR-TB^m^	13 (0.7)
Pre-XDR-TB^n^	4 (0.2)
XDR-TB^o^	0 (–)
**Total cases with drug susceptibility** **testing results**	1815 (86.6)^p^

MDR: multidrug-resistant; TB: tuberculosis; XDR: extensively drug-resistant.

^a^ A new case refers to a patient who has never been treated for TB or has been treated previously for < 1 month.

^b^ A relapse case refers to a patient who is diagnosed with TB and has been previously treated (fully or partially) for TB in Australia or overseas.

^c^ Pulmonary TB including other sites refers to any bacteriologically confirmed or clinically diagnosed case of TB involving the lung parenchyma or the tracheobronchial tree.

^d^ Extra-pulmonary TB refers to any bacteriologically confirmed or clinically diagnosed case of TB involving organs other than the lungs, for example, pleura, lymph nodes, abdomen, genitourinary tract, skin, joints and bones, and meninges. More than one extra-pulmonary site may be reported for each notified case of TB.

^e^ Bacteriologically confirmed TB is confirmed through laboratory diagnosis. (*15*) A bacteriologically confirmed TB case is one in which a biological specimen is positive by smear microscopy, culture or WHO-approved rapid diagnostics (such as Xpert® MTB/RIF assay).

^f^ Drug susceptibility testing here refers to cases that are culture-confirmed.

^g^ Excludes cases with no reported risk factors. More than one risk factor may be reported for each notified case of TB. Risk factor information for acquiring TB is collected at the time of diagnosis by the relevant health departments or medical practitioners.

^h^ A high-risk TB country is defined by the Australian Government Department of Home Affairs and National TB Advisory Committee as a country with an annual TB incidence > 60 cases per 100 000 population. (*18*)

^i^ Totals do not add up to 100% as cases may be counted across multiple resistance categories.

^j^ First-line anti-TB agents are rifampicin, isoniazid, ethambutol and pyrazinamide.

^k^ Second-line injectable anti-TB agents are kanamycin, capreomycin and amikacin.

^l^ Fluoroquinolones are ciprofloxacin, ofloxacin, moxifloxacin and levofloxacin.

^m^ Multidrug-resistant TB is resistant to at least isoniazid and rifampicin but not XDR-TB.

^n^ Pre-XDR-TB is resistant to isoniazid, rifampicin and a fluroquinolone

OR isoniazid, rifampicin and a second-line injectable (amikacin, capreomycin, kanamycin).

^o^ XDR-TB is resistant to isoniazid and rifampicin, and any of the fluoroquinolones, and to at least one of the three injectable second-line drugs.

^p^ From a total of *n* = 2095.

The majority of TB cases were bacteriologically confirmed (90.6%), of which 88.5% (*n* = 1789) were confirmed by culture, and 98.2% (*n* = 1756) of isolates underwent drug susceptibility testing (**Table 2**). Drug susceptibility testing results were available for 86.6% (1815/2095) of all elderly TB cases, with isoniazid mono-resistance found in 4.2% (*n* = 77), rifampicin mono-resistance in 0.3% (*n* = 6), multidrug resistance (MDR) in 0.7% (*n* = 13) and pre-extensive drug resistance (pre-XDR) in 0.2% (*n* = 4) (**Table 2**).

Unsuccessful treatment, which included those with treatment failure, default on treatment, and death while on treatment, was reported in 6.9% of cases (*n* = 156) (**Table 1**). Among these, 8.7% (*n* = 10) were relapse/recurrence cases and one (0.9%) had MDR-TB. A relatively high percentage of elderly cases, 4.9% (*n* = 109), died of TB during treatment, of whom 81.7% (89/109) were born overseas, 88.9% (97/109) were newly diagnosed, 88.9% (97/109) had pulmonary TB, 39.4% (43/109) were ≥ 85 years of age and 0.9% were pre-XDR (1/109).

In both univariate and multivariable analyses, the most important risk factor influencing treatment success was age, with more favourable outcomes in younger age groups (**Table 3**). The odds of treatment success in cases who had a history of travel or residence in a country with high TB incidence were nearly twice that of cases who did not. The odds of treatment success with those diagnosed ≥ 10 years after their arrival in Australia were 1.5 times greater than those who were diagnosed < 10 years after arrival (**Table 3**).

**Table 3:** Univariate and multivariable analyses of factors associated with TB treatment success, Australia, 2011–2020

Variable	Univariate analysis treatment outcome (*n* = 1 755)^a^	Multivariable analysis treatment outcome (*n* = 1 752)^a,b,c^
	Crude OR (95% CI)	Adjusted OR (95% CI)
**Demographics**		
Male sex	0.77 (0.54–1.11)	–
Female sex	1.28 (0.89–1.82)	–
Age groups (years)		
65–69	**3.72 (2.15–6.39)**	**5.5 (3.14–9.81)**
70–74	**2.16 (1.32–3.54)**	**3.61 (2.14–6.08)**
75–79	0.94 (0.62–1.41)	**1.79 (1.16–2.77)**
80–84	**0.52 (0.35–0.76)**	–
≥ 85	**0.35 (0.24–0.49)**	–
Aboriginal and/or Torres Strait Islander	0.61 (0.18–2.10)	–
Born in Australia	0.67 (0.44–1.01)	–
Born overseas	1.48 (0.98–2.25)	–
**Diagnosis**		
Pulmonary TB (including other sites)	1.01 (0.69–1.47)	–
HIV-positive (coinfection)	0.48 (0.06–4.19)	–
**Treatment history**		
Any previous TB treatment	1.16 (0.61–2.19)	–
**Drug susceptibility**		
Fully susceptible	1.03 (0.72–1.47)	–
Resistance to first-line anti-tuberculosis agents	0.95 (0.53–1.69)	–
Resistance to second-line injectables	0.19 (0.02–2.15)	–
Resistance to fluoroquinolones	**0.10 (0.01–0.69)**	**0.11 (0.01–0.78)**
Multidrug-resistant TB	0.87 (0.11–6.97)	–
Pre-extensively drug-resistant TB	0.29 (0.30–2.82)	–
**Risk factors for TB**		
Past travel to or residence in a country with high TB incidence	**1.80 (1.26–2.58)**	–
Household or other close contact with TB	1.01 (0.63–1.85)	–
Currently receiving immunosuppressive therapy	0.96 (0.51–1.84)	–
Chest X-ray suggestive of old untreated TB	1.74 (0.69–4.36)	–
Employed in the health industry in Australia or overseas, currently or in the past 5 years	0.86 (0.31–2.45)	–
Resided in an aged-care facility within the past 5 years	**0.14 (0.07–0.30)**	**0.24 (0.11–0.52)**
Resided in a correctional facility within the past 5 years	0.97 (0.12–7.68)	–
Homeless within the past 5 years	0.68 (0.08–5.58)	–
Time from arrival to diagnosis		
< 2 years	**0.50 (0.35–0.71)**	**0.48 (0.34–0.69)**
2–4 years	5.91 (0.81–42.93)	–
5–9 years	1.49 (0.72–3.11)	–
≥ 10 years	**1.45 (1.04–2.02)**	–

Bold values are statistically significant (*P* < 0.05).

CI: confidence interval; OR: odds ratio; TB: tuberculosis.

^a^ Univariate and multivariable analysis binary outcome comparison groups: “variable (ref: reference group variable)”; for males (ref: non-males); females (ref: non-females); each 5-year age group (ref: all other elderly age groups combined); Aboriginal and/or Torres Strait Islander (ref: non-Aboriginal or Torres Strait Islander); born in Australia (ref: not born in Australia); born overseas (ref: not born overseas); pulmonary TB (ref: non-pulmonary TB); HIV-positive (ref: non-HIV-positive); previous treatment (ref: no previous treatment);

resistance to first-line TB agents (ref: no resistance to first-line TB agents); resistance to second-line injectables (ref: no resistance to second-line injectables); resistance to

fluoroquinolones (ref: no resistance to fluoroquinolones); multidrug-resistant TB (ref: non-multidrug-resistant TB); each risk factor category (ref: all other risk factors combined); and time (years) since arrival to diagnosis (ref: all other time since arrival of groups combined).

^b^ Total observations were 1752 for the multivariable model due to three unknowns from time of arrival to diagnosis.

^c^ Odds were adjusted for three 5-year age groups, resistance to fluoroquinolones, ever residing in an aged-care facility, and from time of arrival to diagnosis (0–2 years).

The multivariable analysis found treatment success was significantly associated with the 65–69-year, 70–74-year and 75–79-year age groups compared with all other age groups among elderly cases. Risk factors associated with poor TB treatment outcome included being ≥ 80 years of age with resistance to fluoroquinolones, having resided at any time in an aged-care facility, and being diagnosed within 2 years of arrival in Australia (**Table 3**).

## Discussion

Cases of TB in elderly people are not a major contributor to the Australian TB burden, and elderly patients are not at significantly higher odds of developing TB compared to younger age groups. The epidemiological features of TB cases are broadly similar across elderly and non-elderly age groups. Our findings contrast with most other low-burden countries in the Western Pacific Region, where the highest burden of TB occurs among elderly people (≥ 65 years) with disease rates linked to increased longevity. (*5*, *19*) Although the notification rate is slightly higher among older age groups in Australia, it is not increasing over time and the highest disease burden is occurring among those aged 15–44 years. Most elderly cases in Australia were born overseas and/or had a history of past travel to or residence in a high-incidence country, and ageing likely contributed to reactivation of LTBI.

Migrants from countries with high TB incidence in South and central Asia (*14*, *20*) accounted for 85% of elderly cases in Australia. Increases in the notification rate of TB in Australia and in other low-incidence countries are strongly influenced by migration flows and population growth. (*20*-*25*) Elderly migrants from Cambodia, China, India, the Philippines and Viet Nam contributed a high proportion of TB notifications in Australia, despite the stringent pre-migration screening. Interestingly, the frequency of the top five countries of birth differed between the elderly and non-elderly age groups. China was among the top five countries where migrants in the elderly age group were born, while Nepal was one of the most common countries of birth for migrants in the non-elderly age group. The differences in the top countries of birth across these broad age groups may reflect a relationship with migration patterns, purpose of migration (for example, students or elderly people migrating with family members on permanent visas), offshore pre-migration testing, and the historical TB burden in their country of birth. (*20*, *26*) Even though Australia has a sustained low annual TB incidence rate, (*27*) it is important to note that Australian-born cases represented 15% of all elderly notifications. We hypothesized that individuals were likely to have been exposed to the bacteria during travel to an endemic country or may have acquired LTBI before the 1950s in Australia when the incidence of TB was higher, with over 45 cases per 100 000 population. (*28*) Our findings showed that being born in Australia led to poorer treatment outcomes compared with cases who were born overseas, although these results were borderline significant. Potential reasons for this could include delayed diagnosis, which is suggested by the longer median time from first health presentation to diagnosis compared with people born overseas.

Migrants to Australia and long-term visa holders from TB-endemic countries may be at increased risk of disease during their lifetime, due to a greater likelihood of travel to and extended stays in their country of origin. A study in the United States of America found that children were at increased risk of TB from travel to high-incidence countries or exposure to household visitors from these settings. (*29*) Similar to previous findings, common risk factors associated with TB in elderly people included past travel to or residence in a high-incidence country or close contact with an active TB case. (*11*, *27*) Outbreaks may occur among migrant communities due to different living conditions upon arrival that increase their vulnerability, extensive social networks, and co-residence with multiple generations. (*14*, *30*-*32*) The higher frequency of TB in patients born overseas may also reflect the barriers migrants face, including discrimination, fear of deportation, as well as language, social support, health literacy challenges, and access to free and timely health care. (*24*) The median time from migration to diagnosis with TB in Australia was 25 years. Research from the United Kingdom of Great Britain and Northern Ireland suggested that early case detection is improved with the implementation of catch-up screening 4 years after migration from a country with high TB incidence. (*21*) However, significant evidence gaps still remain around effective approaches to LTBI screening and management in migrants. (*13*) As outlined in a TB elimination framework for low-incidence settings, to overcome migrant health-care and treatment barriers, migrant countries must incorporate culturally and socially appropriate strategies into their health services. (*24*) the Netherlands has documented treatment success in over 90% of migrants through TB policies that enable access to health care through social support. (*33*) Given the disproportionate number of TB cases among migrants, TB services in Australia may need to consider prioritizing earlier or systematic health-services support for elderly migrants from high-incidence countries.

Ageing is a major contributor to TB reactivation risk due to a large burden of undiagnosed LTBI in elderly people, (*34*-*36*) which represents an important reservoir of TB infection. (*14*, *37*) In Hong Kong Special Administrative Region SAR (China), Japan and Singapore, the TB epidemic is largely driven by reactivation of disease due to age-related immune senescence and the prevalence of comorbidities. (*38*) An Australian study found a low (5.1% in 2016) but increasing prevalence of LTBI, with the largest proportion among overseas-born residents aged ≥ 65 years. (*34*) In our study, most cases of TB also represented likely LTBI reactivation given that most have been in Australia for an extended period of time or did not have recent known TB contact. In China, identified TB risk factors among elderly people included age, being male, low socioeconomic status, smoking, previous treatment for TB, and low body mass index (< 18.5). (*39*) Many of these social and lifestyle risk factors are not routinely reported and could not be assessed in our study.

The multivariable analysis showed that greater odds for successful treatment were associated with the ages 65–79 years. The analysis also showed that the odds of unsuccessful treatment were associated with resistance to fluoroquinolones, residing in an aged-care facility, and being diagnosed within 0–2 years of arrival in Australia. Residing in an aged-care facility is an important risk factor for people from high-income countries to be exposed to TB, (*36*, *38*) but there is limited evidence regarding treatment outcomes and residing in these settings. Contrary to expectations, being diagnosed earlier (0– < 2 years) was associated with poorer treatment outcomes. This may be due to several reasons, for example, a more clinically advanced or severe infection, a lack of culturally appropriate health services or a specialist migrant health workforce. There may also be individual barriers including different health-seeking behaviours, health literacy, physical access to health-care facilities, and linguistic skills, which may impact treatment compliance and continued health-care engagement. (*40*)

The literature suggests that most TB disease in elderly people is due to the reactivation of LTBI, (*10*, *34*-*36*) the treatment for which may be a principal preventive factor for the control of TB among those of advanced age. Historically, elderly people have not been prioritized for LTBI treatment due to a higher risk of adverse events. (*41*) However, in recent years, shorter rifamycin-containing regimens have also been recommended, with a lower risk of toxicity. (*41*) Further research to support the efficacy of this approach including informing elderly people of the risk–benefit ratio of TB preventive therapy is warranted. (*42*)

A strength of our study is that it used a comprehensive national TB data set. The backward stepwise approach to the multivariable model enabled us to exclude variables with collinearity and to consider the effects of all variables simultaneously. Australia is a country with low TB incidence, with a universal health-care system providing treatment and care for people with TB for free or with no out-of-pocket expenses, regardless of eligibility for free public health care. Therefore, the results of our analysis will be most relevant to other low-incidence settings with universal health-care systems.

The analyses had several limitations. There were completeness and quality issues for several variables due to changes in reporting over time for risk factor information and HIV status. The NNDSS data set also lacked a treatment completion date, a TB death date, relevant comorbidities, and known lifestyle risk factors, which may have been unknown confounders when assessing treatment success. Our multivariable analysis had high standard errors for the 65–69-year and 70–75-year age groups, representing greater variability and uncertainty in these results. Additionally, the group size of some variables in our multivariable analysis was small (< 10 cases), which may have potentially led to a reduced association with the outcome of successful treatment.

Our findings showed that elderly people represent only a small proportion of all TB cases reported in Australia. Similar to non-elderly age groups, most elderly cases are migrants from countries with high TB incidence. Australia has committed to working towards the elimination of TB by 2035. A critical component to achieving this goal will be the prioritization of the needs of our migrant population and identifying optimal ways to reduce LTBI reactivation. TB elimination in low-incidence settings is contingent upon the diagnosis and treatment of LTBI, as per the WHO End TB Strategy. In line with the National TB Advisory Committee strategic plan (2021–2025), migrants are a critical group for the prevention of TB and the reduction of its incidence in Australia. Our findings have demonstrated that the majority of elderly TB cases in Australia are migrants and an unknown number of these could have reactivated LTBI. Additional research into the LTBI reactivation risk and LTBI treatment among this group would be valuable to explore this possibility. The risk-benefit ratio of these interventions in Australia has not been fully established.

Australia has well functioning jurisdictional TB control programmes that limit secondary cases and local transmission of TB, which is essential in maintaining and continuing to reduce Australia’s low incidence of TB. Further exploration of the elderly population could be undertaken to investigate the differences between non-elderly and elderly TB cases, the relationship between risk factor information and treatment outcomes, risk factors for LTBI in migrants, and predictors for unsuccessful treatment in elderly cases.
